# Exploring the Caregiving Context of Memory Challenges in Both Care Recipients and Care Partners

**DOI:** 10.1093/geroni/igaf122.3584

**Published:** 2025-12-31

**Authors:** Sophie Park, Jessica Hahne, Ella Kohn, Kejia Zhang, Sara Czaja, Megan Shen, Kelly McConnell

**Affiliations:** Weill Cornell Medicine, New York, New York, United States; Washington University in St. Louis, St. Louis, Missouri, United States; Weill Cornell Medicine, Weill Cornell Medicine, New York, United States; Weill Cornell Medicine, Weill Cornell Medicine, New York, United States; Weill Cornell Medicine, New York, New York, United States; Fred Hutchinson Cancer Center, Seattle, Washington, United States; Memorial Sloan Kettering Cancer Center, Memorial Sloan Kettering Cancer Center, New York, United States

## Abstract

Many care partners (CPs) of care recipients (CRs) with memory challenges also experience cognitive challenges. While the role of “care partner” carries the assumption of intact cognition, there is limited research examining memory challenges among CPs. In a pilot study of an advance care planning intervention for CRs with memory challenges and their CPs, 16 unpaired CRs and CPs and 21 CR-CP dyads were screened for study eligibility with the Montreal Cognitive Assessment-Blind (MoCA-B). Descriptive statistics were used to examine CP scores. Audio recordings of the intervention sessions were coded to quantify explicit references to memory challenges among CPs and CRs serving dual roles as a CR & CP. Five CPs had MoCA-B scores indicating cognitive impairment (MoCA-B< 19; M = 17, SD = 1.22). MoCA-B scores were identical for the CR and CP in three dyads (Range=18-19). In two dyads, the CR had a higher MoCA-B score than the CP (CR Range=18-21, CP Range=17-18). The results also identified two CRs who reported being a CP in another caregiving relationship. Additionally, four of nine CPs reported that they experience memory challenges and described reciprocal caregiving relationships where they received help from their CRs. These findings challenge the prevailing assumption that CPs are fully prepared to meet caregiving demands and suggest that there is significant variability in the cognitive capacity of CPs. Overlooking these differences may underrepresent the challenges CPs face and the support they may require. There is further need to address this issue to accurately understand care dynamics and develop effective care models.

